# Supplementary material for: A computational Evo-Devo approach for elucidating the roles of PLETHORA transcription factors in regulating root development
Source: PLoS One. 2025 Jul 31;20(7):e0327511. doi: 10.1371/journal.pone.0327511 (PMC12312886; doi:10.1371/journal.pone.0327511)
Supplement: S1 Data — (PDF) [file pone.0327511.s007.pdf]

S1 data. Identification of putative *PLETHORA* orthologs of Cactaceae

Cactaceae systematics is often challenging, and while it is somewhat robust at the subfamily level, genus and species or infraspecies relationships change frequently. This is in part because genomic resources for Cactaceae are scarce, with only two draft genomes reported: those of *Carnegiea gigantea* (Copetti et al., 2023) and *Hylocereus undatus* (now *Selenicereus undatus*) (Zheng et al., 2021). In addition, five low-coverage and highly fragmented genomes are publicly available for the species *Lophocereus schottii, Stenocereus thurberi, Pachycereus pringlei, Pereskia humboldtii* (Copetti et al., 2017), and *Cereus fernambucensis* (Amaral et al., 2021). Therefore, most molecular systematic studies of Cactaceae have been performed using plastid or mitochondrial markers, whose evolutionary rates and inheritance patterns differ from those of nuclear genes. As a consequence, assigning orthology relationships between genes is difficult and often leads to mistakenly choosing paralogs instead of orthologs across species.

To overcome this problem, we identified putative orthologs of the five PLTs previously annotated in the root apex transcriptome of *P. pringlei* (Rodriguez-Alonso et al., 2018) in the genomes of *C. gigantea*, *L. schottii*, *S. thurberi*, *H. undatus*, and *P. pringlei* (Copetti et al., 2017, Zheng et al., 2021). All these species belong to the largest Cactaceae subfamily, Cactoideae, which includes ca. 80% of Cactaceae species; within Cactoideae, *H. undatus* is from the Hylocereeae tribe, while the four other species are members of the Pachycereeae tribe. We also compared the identity percentages of *PLT* coding sequences (CDSs).

To identify the putative *PLT* orthologs in cacti, a BLASTn search was conducted for each *P. pringlei PLT* transcript: *PLT1/2a, PLT1/2b, PLT3/7a, PLT3/7b,* and *PLT4* as queries. Hits with an e-value $<1x10^{-50}$ were considered to be putative *PLT* orthologs. The putative 5' and 3' untranslated regions (UTRs) and the CDS were inferred from pairwise alignments of each scaffold with its respective *PLT* ortholog contig from the *P. pringlei* transcriptome.

The gene models of the cactus *PLT* genes are shown below: *Ppr*, *Pachycereus pringlei*; *Cgi*, *Carnegiea gigantea*; *Lsc*, *Lophocereus schottii*; *Sth*, *Stenocereus thurberi*; *Hun*, *Hylocereus undatus*. *Arabidopsis thaliana* (*Ath*) *PLT* genes were extracted from the Phytozome v13 database (https://phytozome-next.jgi.doe.gov/) and are shown for comparison. A 2 kilobase region upstream of the inferred putative transcription start site (TSS) is also shown as red rectangle, when available.

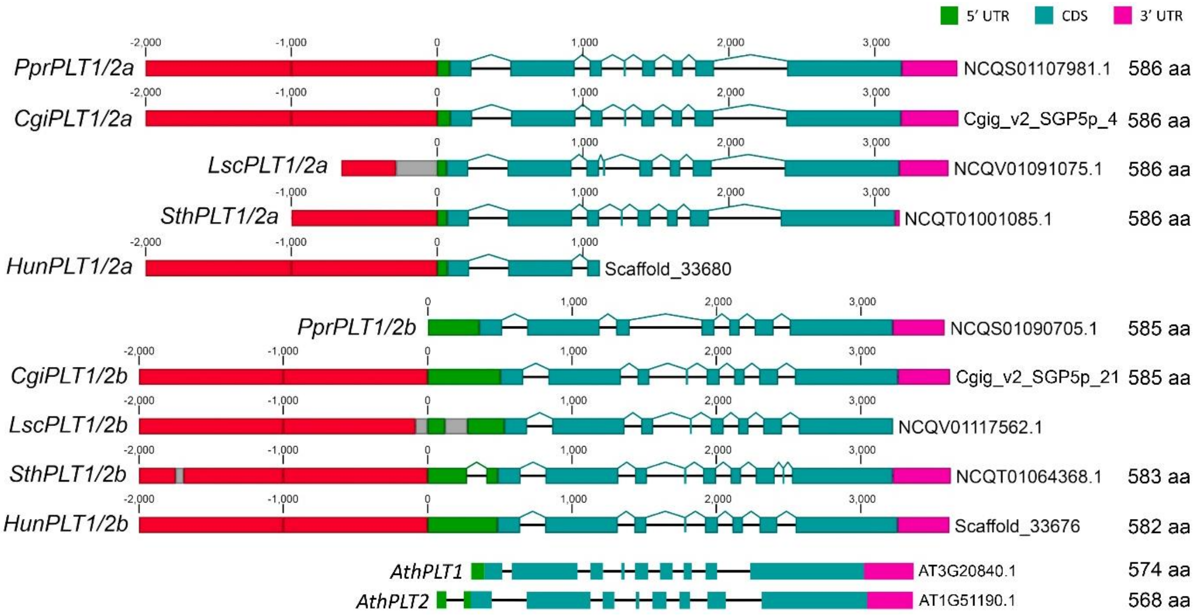

Putative Cactaceae orthologs of *PprPLT1/2a* and *PprPLT1/2b,* two genes from the PLT1-PLT2 clade, share similar exon numbers and lengths with their *Arabidopsis thaliana* counterparts. The intron lengths are also conserved in the genomic sequences of every group of Cactaceae orthologs, *PLT1/2a* and *PLT1/2b*. The scaffold identifiers and lengths of the encoded protein are shown on the right. Note that scaffolds containing some cactus genes are truncated within the coding sequence, inferred 3'-UTR, or putative promoter region before the 2 kb region. Unresolved nucleotides (Ns) are shown as gray rectangles.

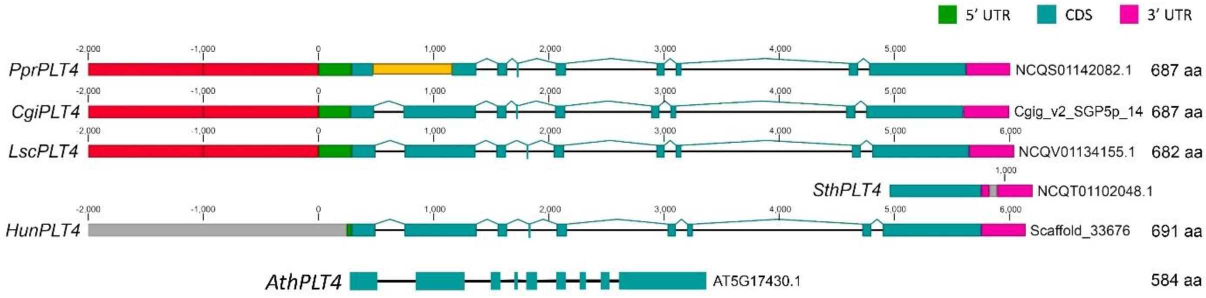

Putative cacti orthologs of *PprPLT4* share similar exon numbers and lengths with *AthPLT4*. The intron lengths are also conserved in the genomic sequences of the Cactaceae orthologs. The scaffold identifiers and lengths of the encoded protein are shown on the right. Note that in the *S. thurberi* scaffold, most of the *SthPLT4* genomic sequence is missing, while part of the *PprPLT4* genomic region failed to align with the *PprPLT4* transcript or the orthologs from all other species (yellow rectangle). The extensive region of *H. undatus* scaffold consists of the unresolved nucleotides (Ns; gray rectangle), which might be a consequence of the sequencing methodology (Illumina short reads and Hi-C).

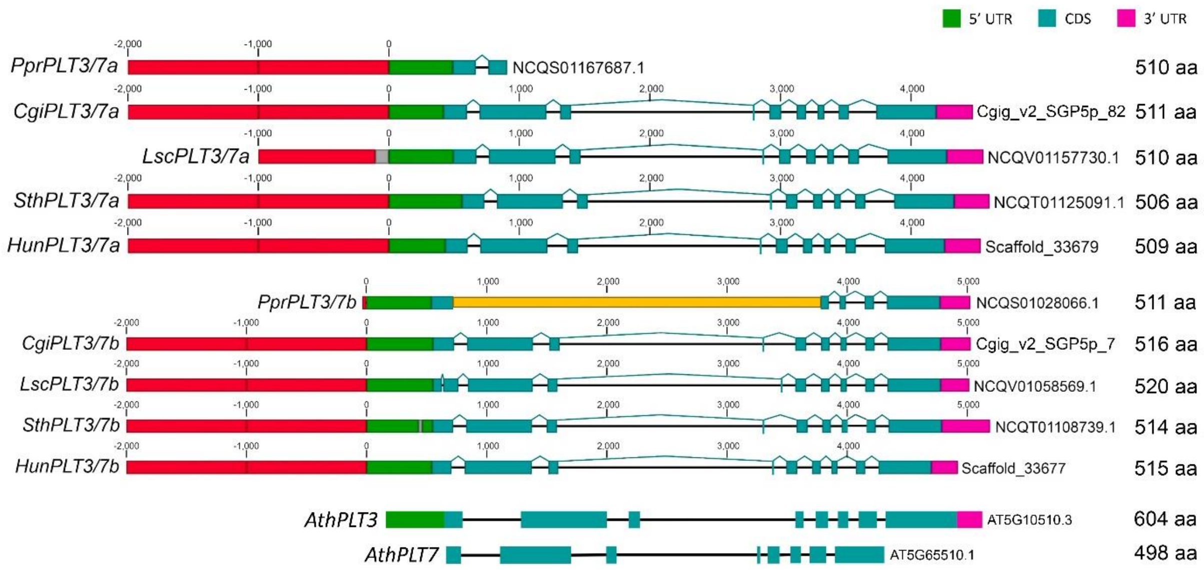

Putative Cactaceae orthologs of *PprPLT3/7a* and *PprPLT3/7b*, two genes from the PLT3 - PLT7 clade, share similar exon numbers and lengths with their counterparts. Intron lengths are also conserved in the genomic sequences of every group of Cactaceae orthologs. Scaffold identifiers and lengths of the encoded protein are shown on the right. Note that the scaffold to which *PprPLT3/7a* was mapped contains only a small region towards the 5' of the CDS, and more than half of the *PprPLT3/7b* genomic region failed to align with the *PprPLT3/7b* transcript or the orthologs from all other species (yellow rectangle), suggesting this genomic fragment was incorrectly assembled.

Next, we analyzed the identity percentage of the CDSs of the possible Cactaceae orthologs. A BLASTn pairwise comparison revealed >94% nucleotide sequences identity; the CDS lengths of the orthologs were identical or very similar:

PLT1/2a	***P. pringlei*** (1,761 nts)	***C. gigantea*** (1,761 nts)	***L. schottii*** (1,761 nts)
C. gigantea (1,761 nts)	98.6		
L. schottii (1,761 nts)	98.2	98.1	
S. thurberi (1,761 nts)	97.6	97.3	96.9

PLT1/2b	***P. pringlei*** (1,758 nts)	***C. gigantea*** (1,758 nts)	***S. thurberi*** (1,752 nts)
C. gigantea (1,758 nts)	98.5		
S. thurberi (1,752 nts)	95.0	94.4	
H. undatus (1,749 nts)	95.5	94.8	95.7

PLT3/7a	***P. pringlei*** (1,533 nts)	***C. gigantea*** (1,536 nts)	***L. schottii*** (1,533 nts)	***S. thurberi*** (1,521 nts)
C. gigantea (1,536 nts)	98.4			
L. schottii (1,533 nts)	97.4	97.4		
S. thurberi (1,521 nts)	96.3	96.2	96.1	
H. undatus (1,530 nts)	96.6	96.6	96.2	96.7

PLT3/7b	***P. pringlei*** (1,536 nts)	***C. gigantea*** (1,551 nts)	***L. schottii*** (1,563 nts)	***S. thurberi*** (1,545 nts)
C. gigantea (1,551 nts)	97.4			
L. schottii (1,563 nts)	96.3	95.4		
S. thurberi (1,545 nts)	97.1	97.1	95.7	
H. undatus (1,548 nts)	95.6	96.0	94.3	96.5

PLT4	***P. pringlei*** (2,064 nts)	***C. gigantea*** (2,064 nts)	***L. schottii*** (2,049 nts)
C. gigantea (2,064 nts)	98.1		
L. schottii (2,049 nts)	97.2	96.9	
H. undatus (2,076 nts)	96.6	96.4	96.2

By contrast, the CDS and intron lengths of the PLT paralogs were different, and their identity percentages were much smaller, as shown here for the CDS and intron lengths *Carnegiea gigantea* paralogs (CDS length is shown on the right):

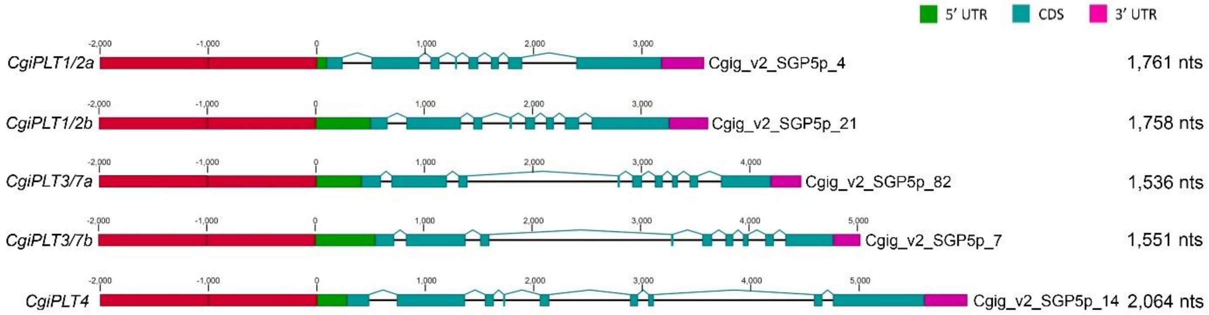

and CDS identity percentages of *Pachycereus pringlei* PLT paralogs (de novo assembled transcripts (32).

	PprPLT1/2a (1,761 nts)	***PprPLT1/2b*** (1,758 nts)	***PprPLT3/7a*** (1,533 nts)	***PprPLT3/7b*** (1,536 nts)
PprPLT1/2b (1,758 nts)	68.8			
PprPLT3/7a (1,533 nts)	50.3	50.0		
PprPLT3/7b (1,536 nts)	50.5	50.8	73.5	
PprPLT4 (2,064 nts)	51.5	53.4	45.1	44.8

Therefore, the CDSs of all putative *PLT* orthologs of five Cactaceae species have similar lengths and share more than 94% identity, while the lengths of the CDSs of the *PLT* paralogs are variable, with identity below 75%.

In conclusion, in the absence of well-assembled genomes (which hindered our implementation of synteny or microsynteny analysis), we showed that gene models could be reconstructed from the available data, including inferring UTRs, intronic regions, and exonic regions, to better assess the orthology relationships of non-model species. In the case of PLTs, which are a particularly difficult to analyze given the duplication of PLT family members, this approach overcomes the need to reconstruct the phylogeny, which could otherwise introduce bias and might mistakenly group sequences due to the inclusion of incomplete protein sequences.
