# Supplementary figures and images for: A computational Evo-Devo approach for elucidating the roles of PLETHORA transcription factors in regulating root development
Source: PLoS One. 2025 Jul 31;20(7):e0327511. doi: 10.1371/journal.pone.0327511 (PMC12312886; doi:10.1371/journal.pone.0327511)

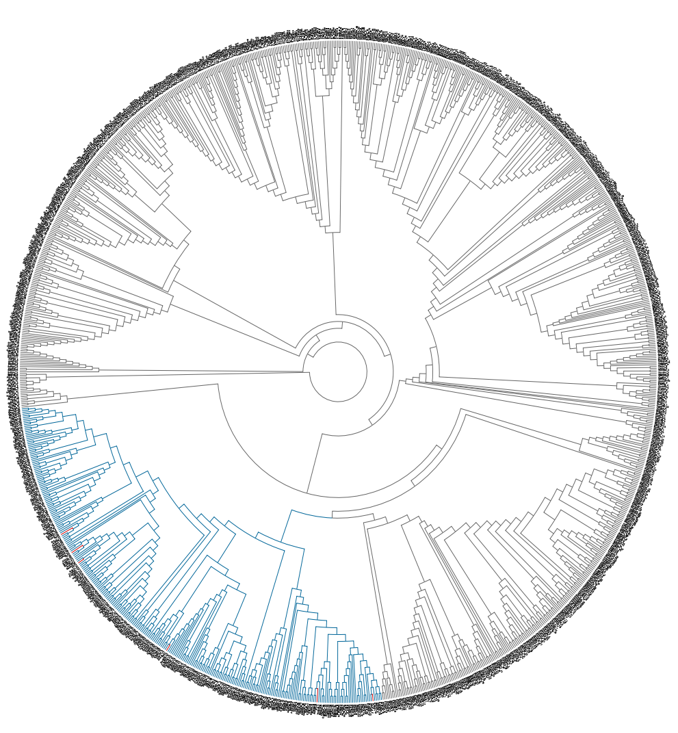

Supplement: S1a Fig — The sequences were retrieved from Viridiplantae genomes as described in the Material and Methods. Only protein sequences that included two consecutive AP2 domains separated by an interdomain linker were used for analysis. All PLT sequences from Arabidopsis thaliana (red branches) are included in a single clade (shown in blue). The complete protein sequences included in this clade were extracted and realigned (referred to as PLT-like sequences). Information about the sequences used to reconstruct this phylogeny, including database identifiers, is provided in S1 and S3 Tables. (TIF) [file pone.0327511.s008.tif]

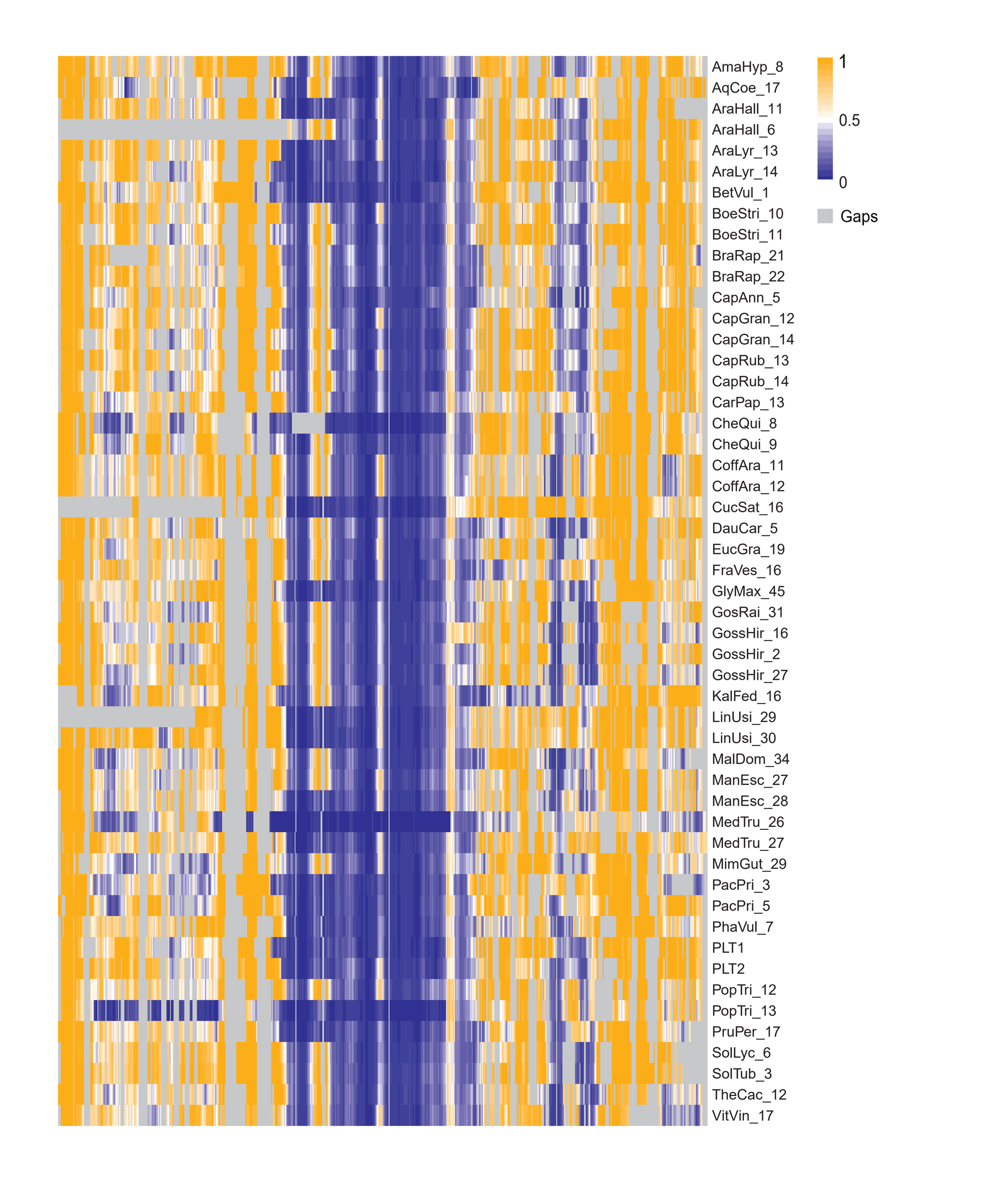

Supplement: S2 Fig — Disorder propensity was analyzed for each PLT sequence of the PLT1-PLT2 clade (Fig 2). Each amino acid in the multiple sequence alignment was substituted by its disorder propensity value. The resulting matrix was visualized as a heatmap. Note that this analysis suggests that the AP2-linker-AP2 region, which corresponds to the DNA binding domains is structured (blue), in line with a crystal structure obtained for a single AP2 domain of TEM1 TF (7ET4) [89]. For complete species names, see S3 Table. (TIF) [file pone.0327511.s010.tif]

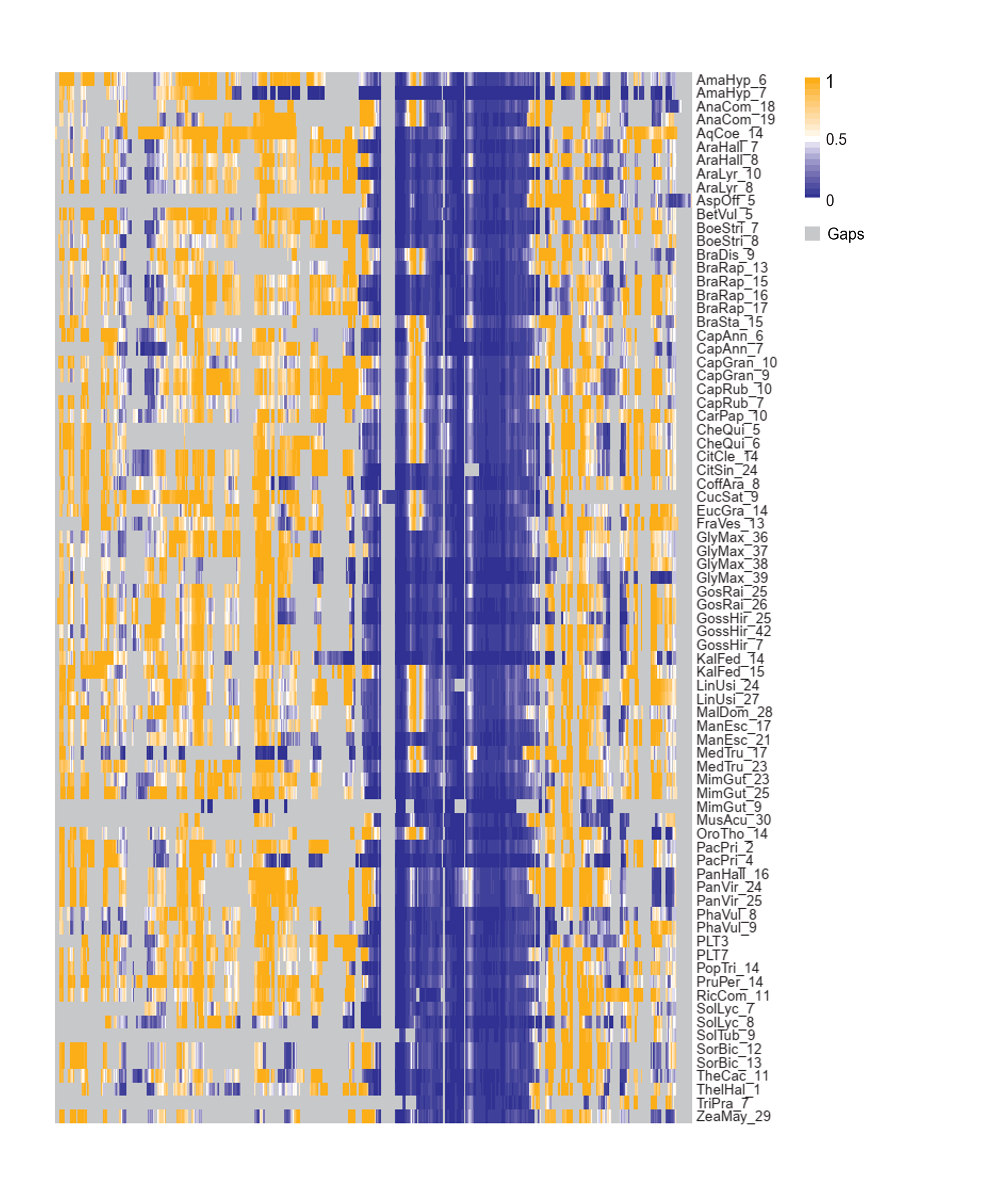

Supplement: S3 Fig — Disorder propensity was analyzed for each PLT sequence of the PLT3-PLT7 clade (Fig 2). Each amino acid in the multiple sequence alignment was substituted by its disorder propensity value. The resulting matrix was visualized as a heatmap. Note that this analysis suggests that the AP2-linker-AP2 region, which corresponds to the DNA binding domains, is structured (blue), in line with a crystal structure obtained for a single AP2 domain of TEM1 TF (7ET4) [89]. For complete species names, see S3 Table. (TIF) [file pone.0327511.s011.tif]

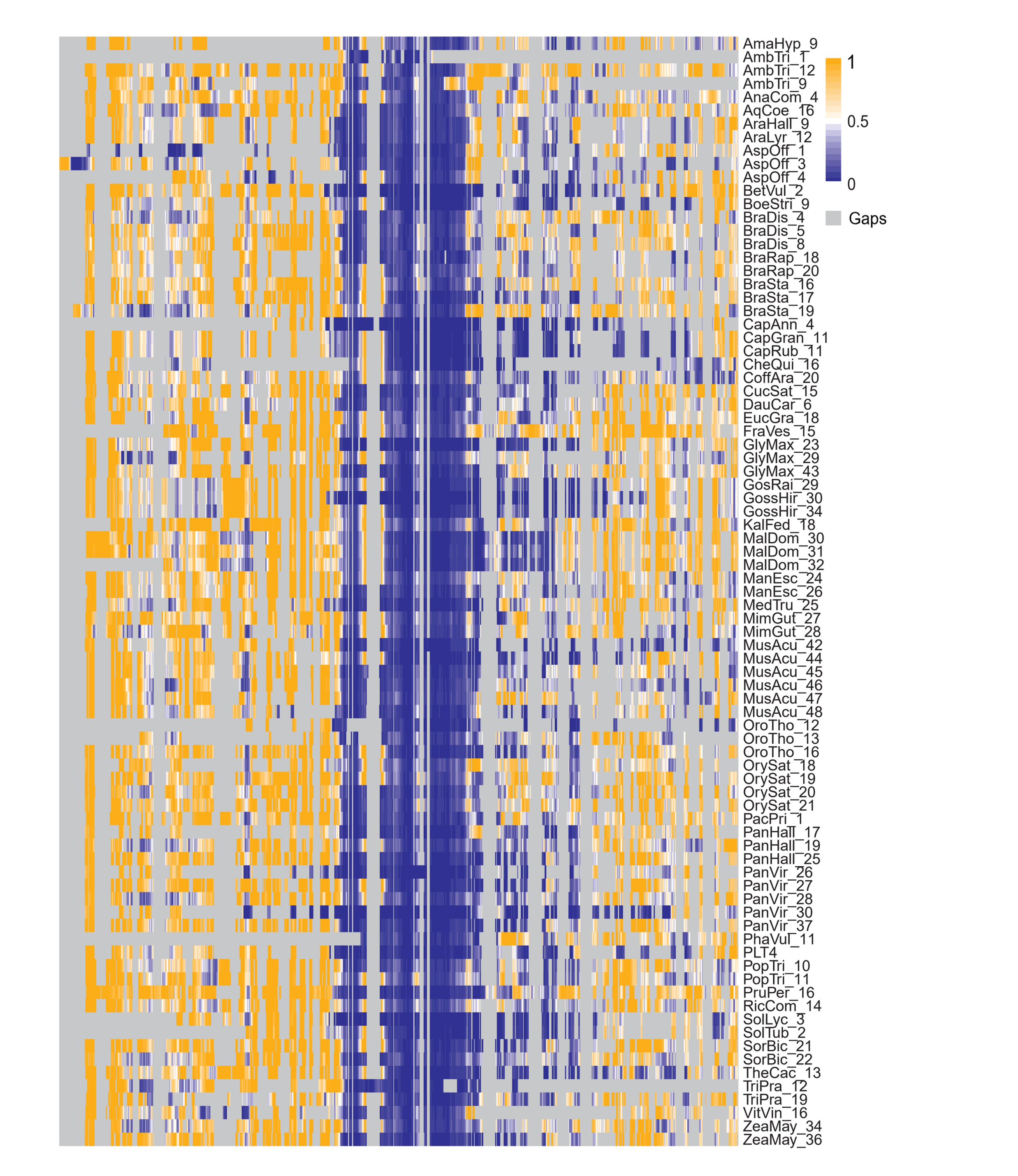

Supplement: S4 Fig — Disorder propensity was analyzed for each PLT sequence of the PLT4 (BBM) clade (Fig 2). Each amino acid in the multiple sequence alignment of the PLT4 clade was substituted by its disorder propensity value. The resulting matrix was visualized as a heatmap. Note that this analysis suggests that the AP2-linker-AP2 region, which corresponds to the DNA binding domains, is structured (blue), in line with a crystal structure obtained for a single AP2 domain of TEM1 TF (7ET4) [89]. For complete species names, see S3 Table. (TIF) [file pone.0327511.s012.tif]

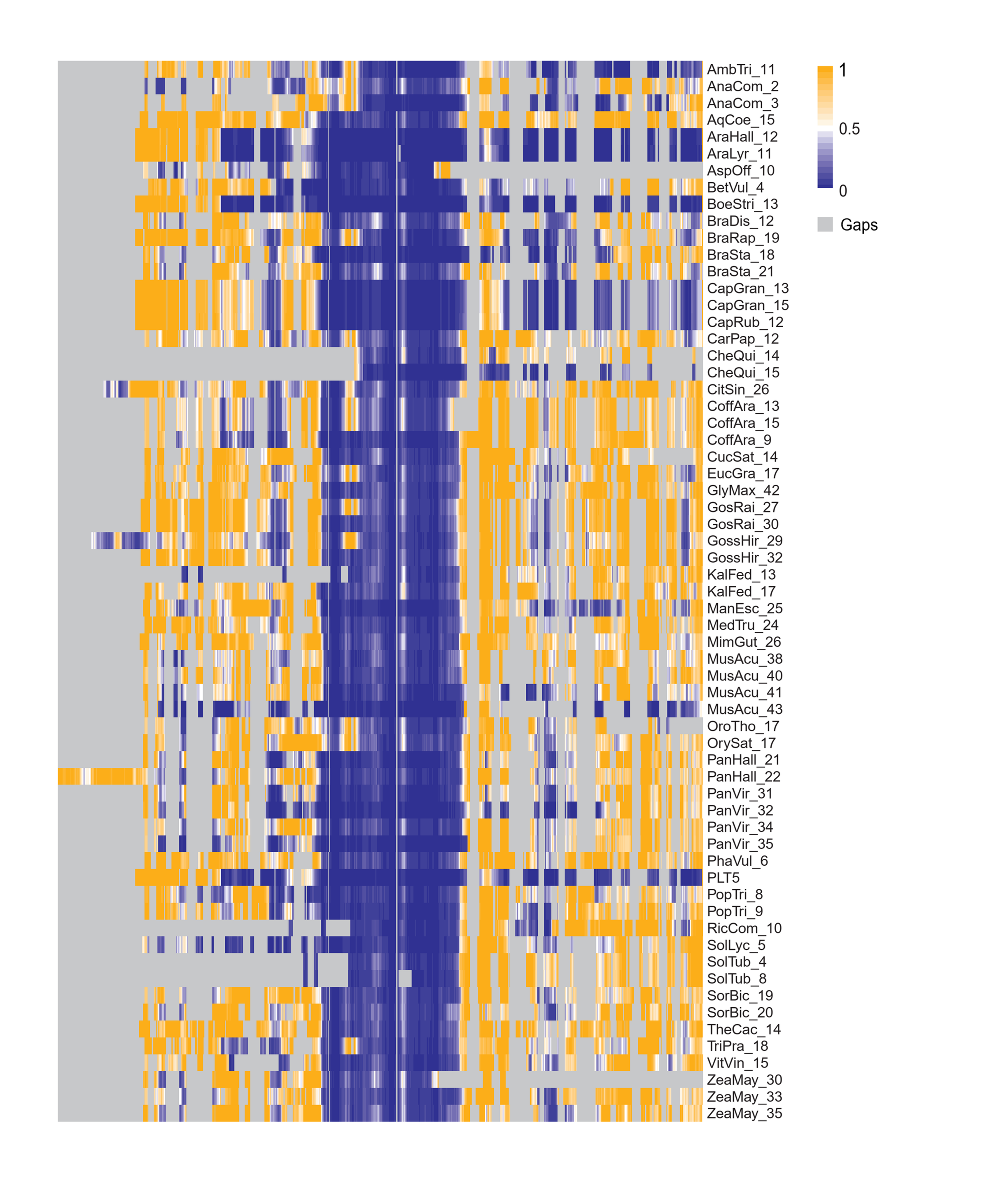

Supplement: S5 Fig — Disorder propensity was analyzed for each PLT sequence of the PLT5 clade (Fig 2). Each amino acid in the multiple sequence alignment of the proteins from the PLT5 clade was substituted by its disorder propensity value. The resulting matrix was visualized as a heatmap. Note that this analysis suggests that the AP2-linker-AP2 region, which corresponds to the DNA binding domains (will be marked in the figures), is structured (blue), in line with a crystal structure obtained for a single AP2 domain of TEM1 TF (7ET4) [89]. For complete species names, see S3 Table. (TIF) [file pone.0327511.s013.tif]

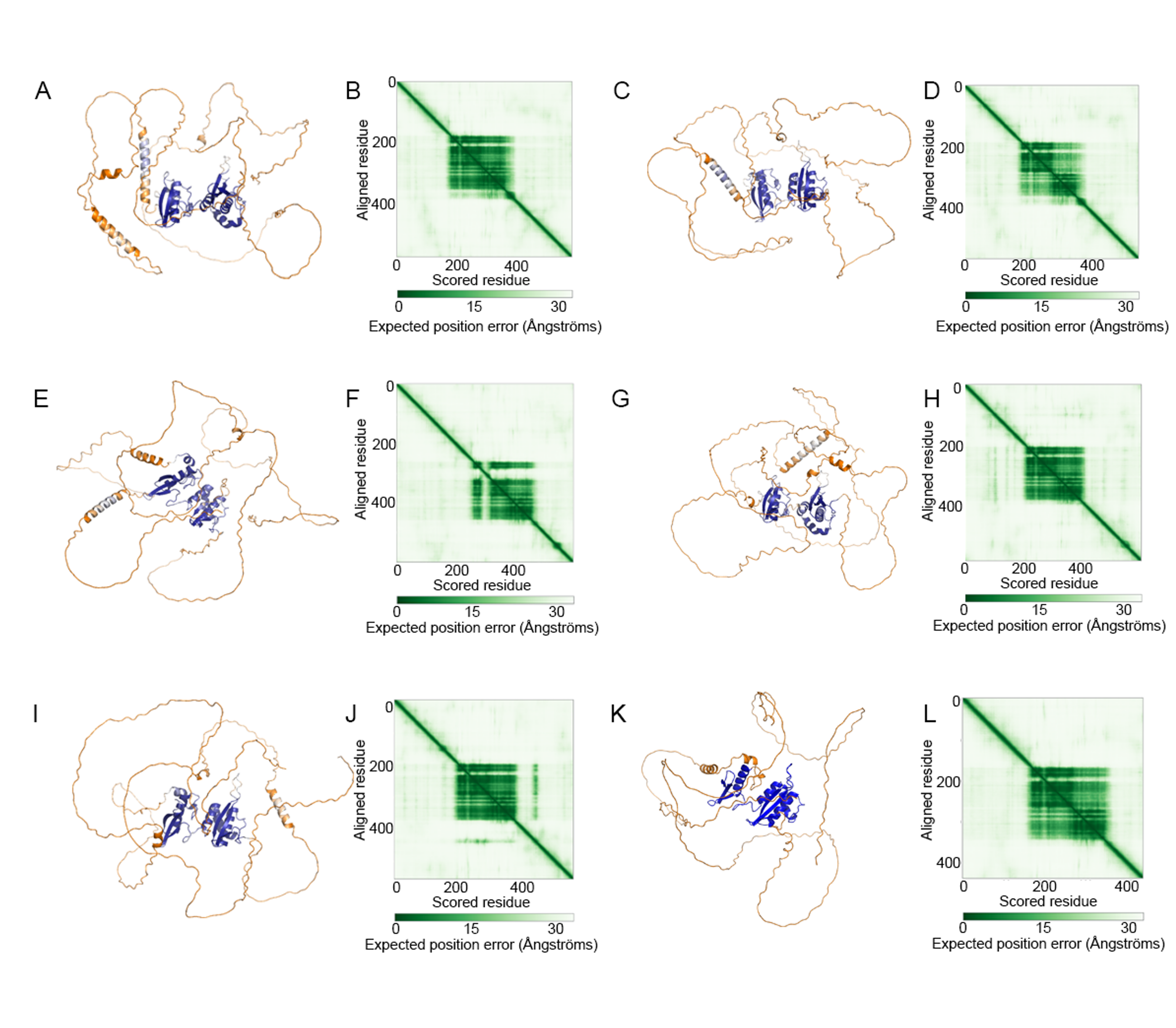

Supplement: S6 Fig — A, C, E, G, I, K: Colors were assigned to the structures according to their disorder propensity values from S2-5 Figures. B, D, F, H, J, L: In agreement with the disorder values, AlphaFold failed to assign a putative structure to regions with higher expected position errors (yellow), supporting the notion that these might be disordered or flexible regions. A, B: PLT1; C, D: PLT2; E, F: PLT3; G, H: PLT4; I, J: PLT5; L, K: PLT7. (TIF) [file pone.0327511.s014.tif]

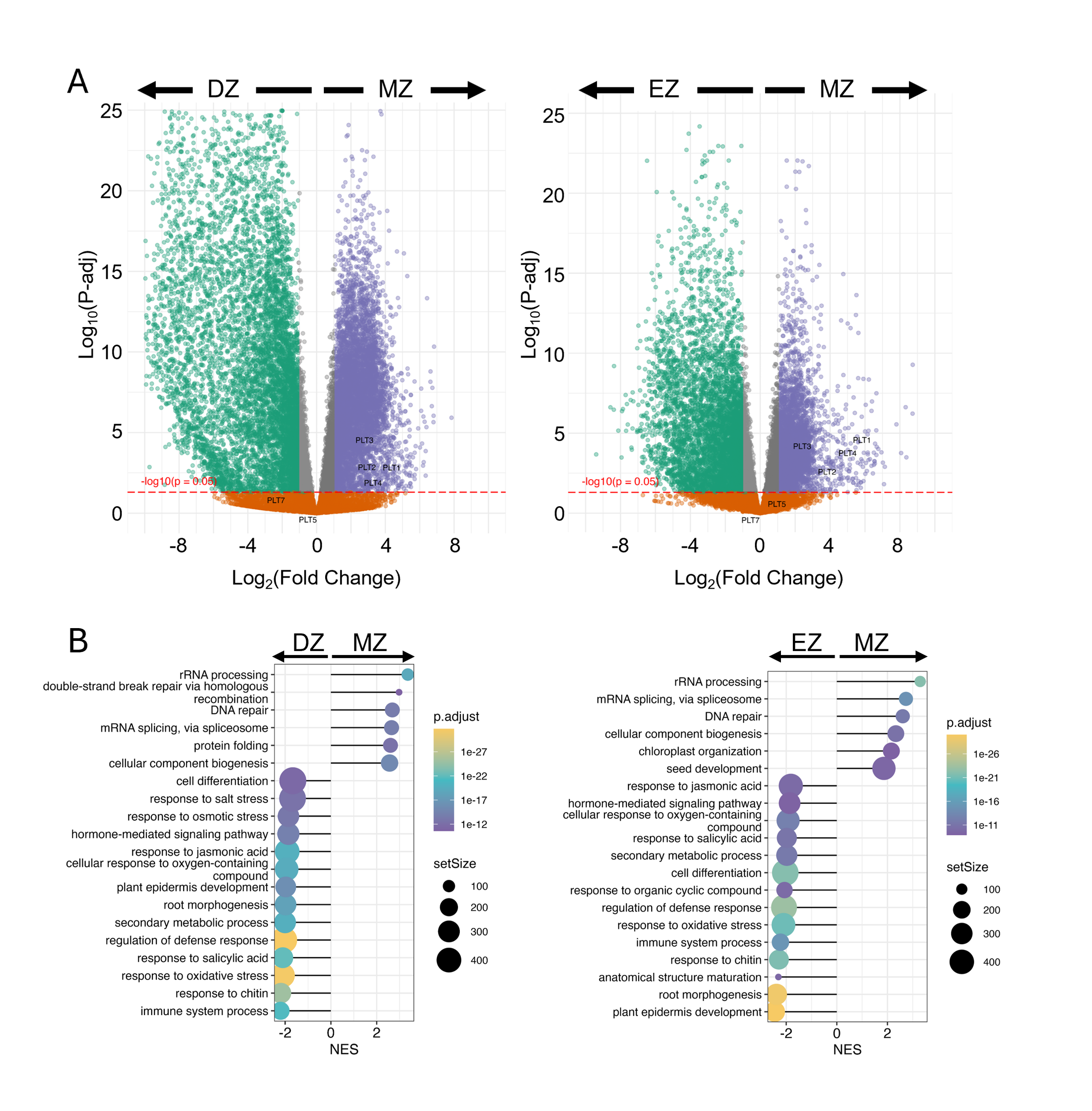

Supplement: S7 Fig — A. Differential gene expression analysis of the RNA-seq data from Huang and Schiefelbein, 2015 [35] performed using DESeq2, fold change ≥2. The set of upregulated genes in the meristematic zone (purple) includes most PLETHORA genes, as labeled in the figure. B. A hypergeometric test based on annotations of the differentially expressed genes in each comparison. NES = Normalized enrichment score. (TIF) [file pone.0327511.s015.tif]

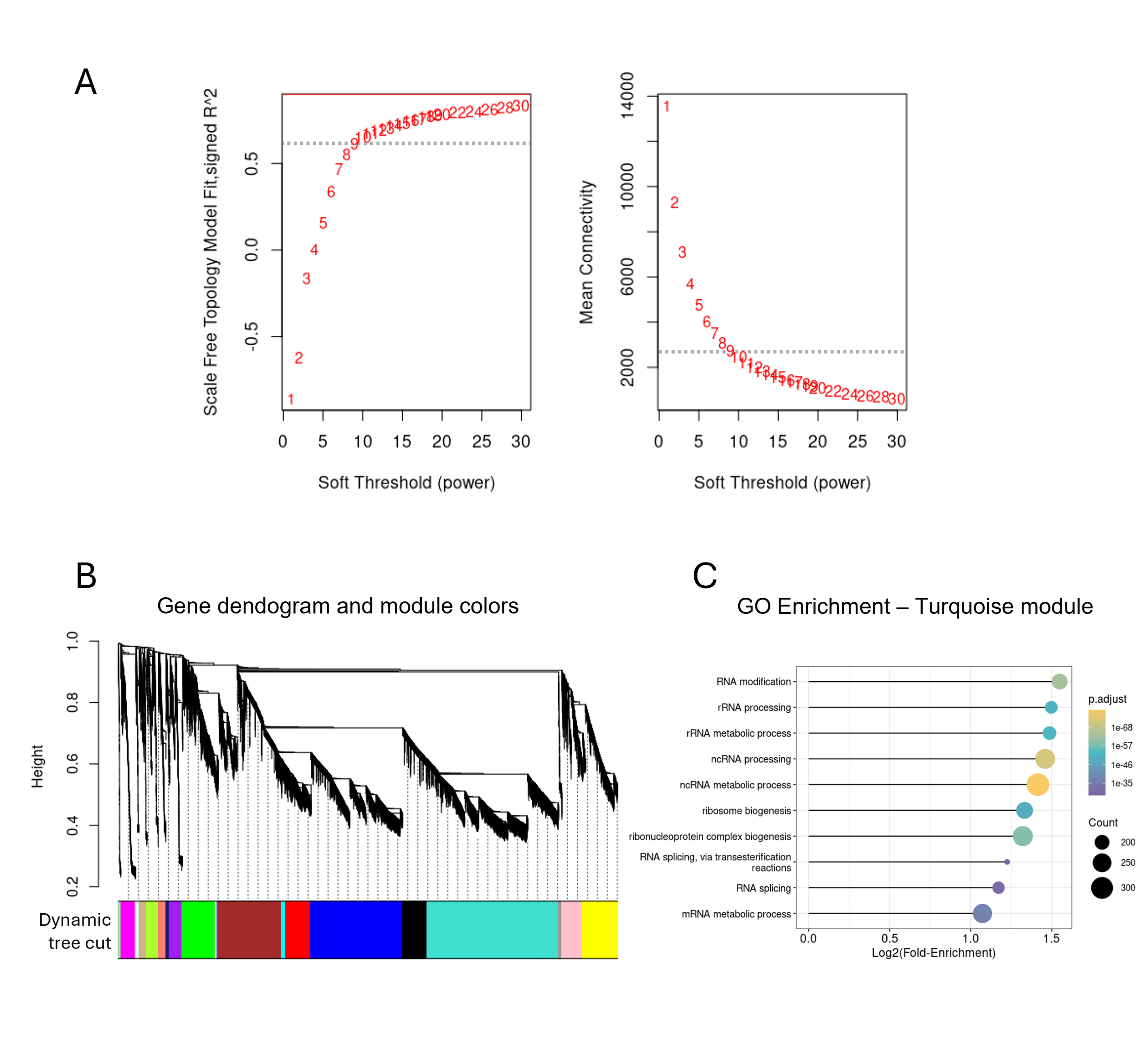

Supplement: S8 Fig — A. Data from A. thaliana meristematic, elongation, and differentiation zones of the primary root generated by Huang and Schiefelbein, 2015 [35] were used for WGCNA. A soft threshold of 9 was used to ensure scale-free dependence. B. The turquoise module, which contains PLETHORA transcription factors, was selected for further analysis. C. The enrichment of biological processes represented by the nodes in the turquoise module shows an overrepresentation of rRNA and ncRNA metabolism, ribosome and ribonucleoprotein biogenesis, and RNA splicing. (TIF) [file pone.0327511.s016.tif]

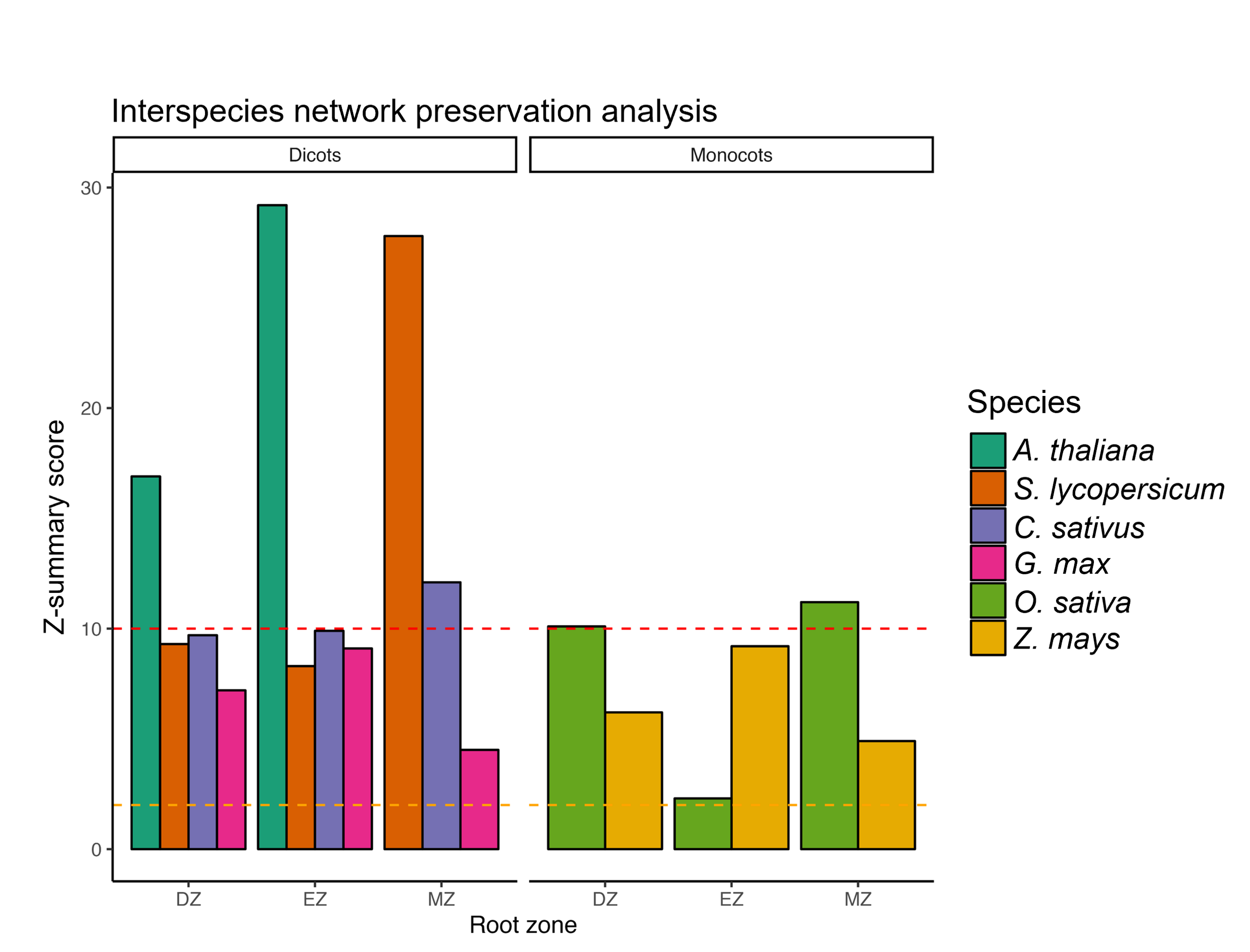

Supplement: S9 Fig — Comparisons of the connectivity and density scores of the WGCNA network for each analyzed species, presented as Z-summary scores. Values >2 suggest evidence of conservation, while values >10 suggest strong evidence of conservation according to Langfelder et al., 2011 [38]. The amino acid sequences of proteins encoded by the genes included in the Arabidopsis thaliana network were used as queries to search for putative orthologs in the analyzed species. (TIF) [file pone.0327511.s017.tif]

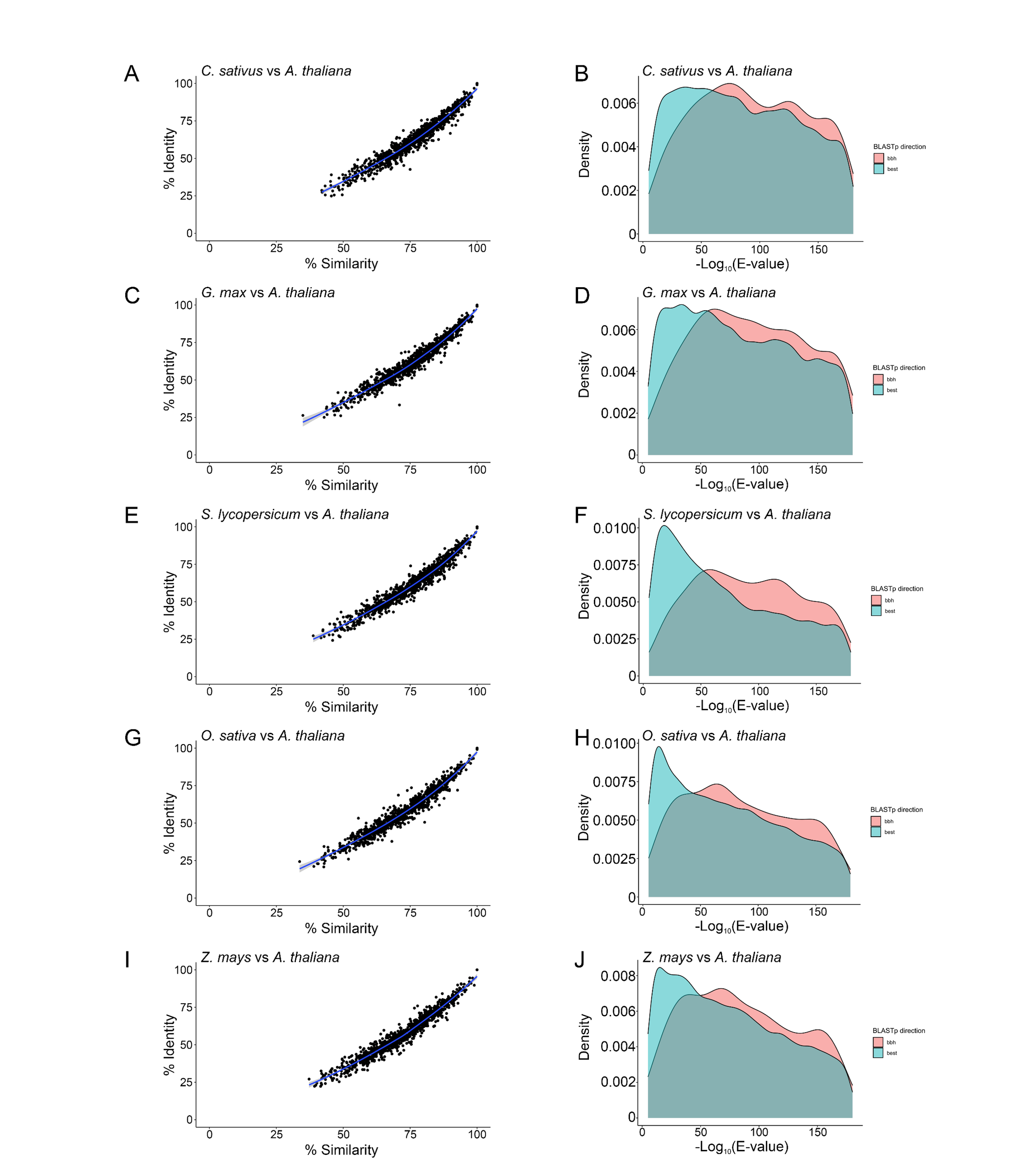

Supplement: S10 Fig — The amino acid sequences of the proteins encoded by the genes in the Arabidopsis thaliana GRN were used as queries to search for putative orthologs in the other species. A, C, E, G, I: Identity and similarity scores between A. thaliana and putative orthologs in each species determined by pairwise BLASTp. B, D, F, H, J: Distribution of e-value for putative orthologs in both best bidirectional hits (bbh) and unidirectional hits (best). (TIF) [file pone.0327511.s018.tif]

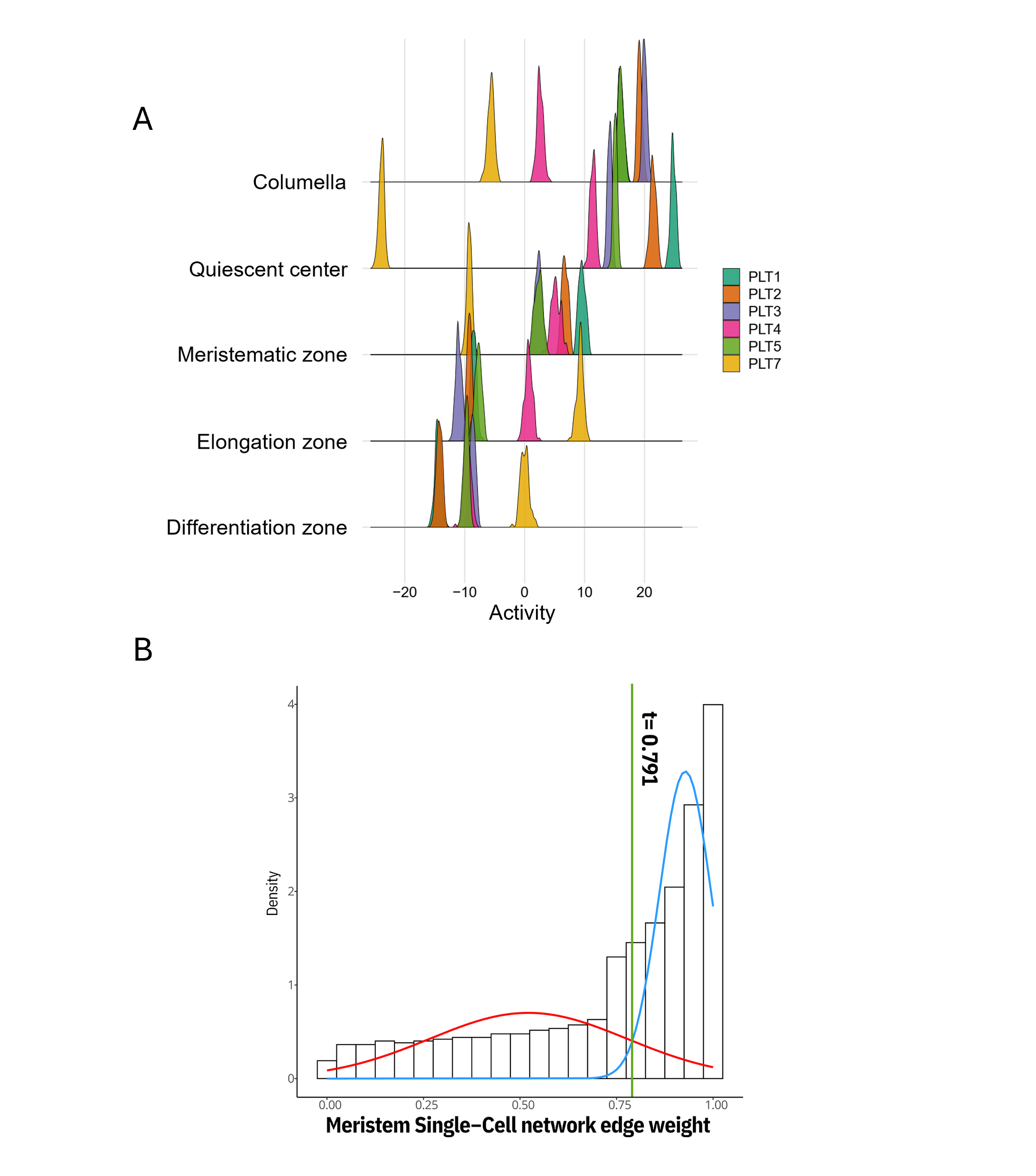

Supplement: S11 Fig — A. The activity (defined as an interpolated and normalized expression profile) of each PLETHORA was calculated to allow cell-type comparisons. Note that PLT1 to PLT4, as well as PLT5, exhibit high positive activity scores in the quiescent center, with the highest score for PLT1 and PLT2. These PLTs also showed high positive activity in the root meristematic zone. B. Gaussian mixture model decomposition analysis to discriminate strong (blue curve) and weak (red curve) interactions in the GRN suggests a threshold of 0.79 for putative biologically relevant interactions (green line). This threshold is used in Fig 5B. (TIF) [file pone.0327511.s019.tif]
